# Effect of Poor Access to Water and Sanitation As Risk Factors for Soil-Transmitted Helminth Infection: Selectiveness by the Infective Route

**DOI:** 10.1371/journal.pntd.0004111

**Published:** 2015-09-30

**Authors:** Adriana Echazú, Daniela Bonanno, Marisa Juarez, Silvana P. Cajal, Viviana Heredia, Silvia Caropresi, Ruben O. Cimino, Nicolas Caro, Paola A. Vargas, Gladys Paredes, Alejandro J. Krolewiecki

**Affiliations:** 1 Instituto de Investigaciones en Enfermedades Tropicales (IIET), Universidad Nacional de Salta–Sede Regional Orán, San Ramón de la Nueva Orán, Salta, Argentina; 2 Consejo Nacional de Investigaciones Científicas y Técnicas (CONICET), Buenos Aires, Argentina; 3 Dirección Nacional de Prevención de Enfermedades y Riesgos, Ministerio de Salud de la Nación, Buenos Aires, Argentina; 4 Fundación Mundo Sano, Buenos Aires, Argentina; 5 Hospital Juan Domingo Perón, Tartagal, Salta, Argentina; Swiss Tropical and Public Health Institute, SWITZERLAND

## Abstract

**Background:**

Soil-transmitted helminth (STH) infections are a public health problem in resource-limited settings worldwide. Chronic STH infection impairs optimum learning and productivity, contributing to the perpetuation of the poverty-disease cycle. Regular massive drug administration (MDA) is the cardinal recommendation for its control; along with water, sanitation and hygiene (WASH) interventions. The impact of joint WASH interventions on STH infections has been reported; studies on the independent effect of WASH components are needed to contribute with the improvement of current recommendations for the control of STH. The aim of this study is to assess the association of lacking access to water and sanitation with STH infections, taking into account the differences in route of infection among species and the availability of adequate water and sanitation at home.

**Methods and Findings:**

Cross-sectional study, conducted in Salta province, Argentina. During a deworming program that enrolled 6957 individuals; 771 were randomly selected for stool/serum sampling for parasitological and serological diagnosis of STH. Bivariate stratified analysis was performed to explore significant correlations between risk factors and STH infections grouped by mechanism of entry as skin-penetrators (hookworms and *Strongyloides stercoralis*) vs. orally-ingested (*Ascaris lumbricoides* and *Trichuris trichiura*). After controlling for potential confounders, unimproved sanitation was significantly associated with increased odds of infection of skin-penetrators (adjusted odds ratio [aOR] = 3.9; 95% CI: 2.6–5.9). Unimproved drinking water was significantly associated with increased odds of infection of orally-ingested (aOR = 2.2; 95% CI: 1.3–3.7).

**Conclusions:**

Lack of safe water and proper sanitation pose a risk of STH infections that is distinct according to the route of entry to the human host used by each of the STH species. Interventions aimed to improve water and sanitation access should be highlighted in the recommendations for the control of STH.

## Author Summary

Soil-transmitted helminths (STH) are a group of parasitic human infections of great public health relevance due to their high prevalence and potentially severe morbidity in case of heavy infection intensity, especially in poor settings. Common species of STH include *Ascaris lumbricoides*, *Trichuris trichiura*, Hookworm (*Ancylostoma duodenale* and *Necator americanus*) and *Strongyloides stercoralis*. Their life cycle includes an obligatory passage on soil, where they mature becoming capable of transmitting the infection to a new host. Two infective routes can be distinguished according to the species, oral ingestion of eggs versus skin penetration of larvae from fecally contaminated soil. Inadequate sanitation and unsafe water supply were described as risk factors for STH infections; however more data on this relationship is needed. The main strategy for STH control is the regular deworming, through mass drug administration. Interventions on water, sanitation and hygiene (WASH) and health education are also important to sustain the benefits of anthelmintic therapy. The present study reports an association between poor sanitation and water access and STH infections selective to the parasite route of entry. This finding could contribute to the design of specific and rational recommendations to reduce soil-transmitted helminths transmission.

## Introduction

Soil-transmitted helminth (STH) infections are the most prevalent neglected tropical disease (NTD) of Latin America and Caribbean region and are considered the most prevalent infection of humankind [1]. Four STH species are of main importance: *Ascaris lumbricoides*, *Trichuris trichiura*, and hookworms (*Ancylostoma duodenale* and *Necator americanus*) [2,3]. *Strongyloides stercoralis* is another STH gaining recognition and starting to be considered in a broader strategy for control [4,5]. STH infections are regarded as cause of iron-deficiency anemia; malnutrition; negative effects on growth and cognitive development of children; adverse outcomes of pregnancy and reduced productivity of adults [6–9]. Northwestern Argentina is a region of known high prevalence of STH [10,11]. Deficient access to sanitation and water are frequent in this region [12,13].

Preventive anthelmintic chemotherapy through mass drug administration (MDA) programs is prioritized by World Health Organization (WHO) as the first line strategy to overcome STH related morbidity [14,15]. In settings with poor access to water, sanitation and hygiene (WASH), the reduction in burden and morbidity of STH infections through MDA is followed by reinfection within a few months after treatment [16,17]. Preventive chemotherapy by itself seems an incomplete strategy to reach control and elimination targets, emphasizing the relevance of interventions intended to reduce environmental exposure to the infective stages of STH [18]. WASH interventions represent a strategy to preserve the benefit of chemotherapy by preventing the uninfected to be exposed, which is sustainable and can eventually lead to transmission interruption and STH eradication [19–22]. Modifications in people’s behavior towards fecally contaminated environments, and improvements in hygiene practices through health education are crucial to reduce transmission of STH [23].

Another issue that should be taken into account among the strategies for STH control is the significant differences in the biology of STH species, which are capable of frustrating a simplistic approach of control that considers STH as a uniform group [24]. Even though the fecally contaminated soil is the common source of transmission for the five species, the route of entry of infective stages from soil to a new host differs. *A*. *lumbricoides* and *T*. *trichiura* infections are acquired when infective eggs are swallowed. While *N*. *americanus*, *A*. *duodenale* and *S*. *stercoralis* infections are acquired through skin penetration of infective third-stage larvae. *A duodenale* and *S*. *stercoralis* larvae are also capable of infecting by ingestion [3,4,25]. Based on their mechanism of entry, STH species can be classified into two groups i) skin-penetrators and ii) orally-ingested. This distinction might be relevant to find specific associations between a risk factor and an infective route. We hypothesized that lacking access to water and sanitation increases the risk of infection with specific STH species, depending on their mechanism of entry. We conducted a cross-sectional study to examine the independent effect of inadequate water supply and sanitation, on the prevalence of STH species grouped by mechanism of entry (skin-penetrators vs. orally-ingested). Data were taken from the baseline phase of a longitudinal survey performed in a high prevalence area of Salta Province, aimed to assess the effectiveness of a community-based MDA program for the control of STH.

## Methods

### Study design

Population based cross-sectional study, conducted in the localities of Tartagal, Orán and Pichanal of Salta Province, Argentina.

### Study population

**Fig 1:**
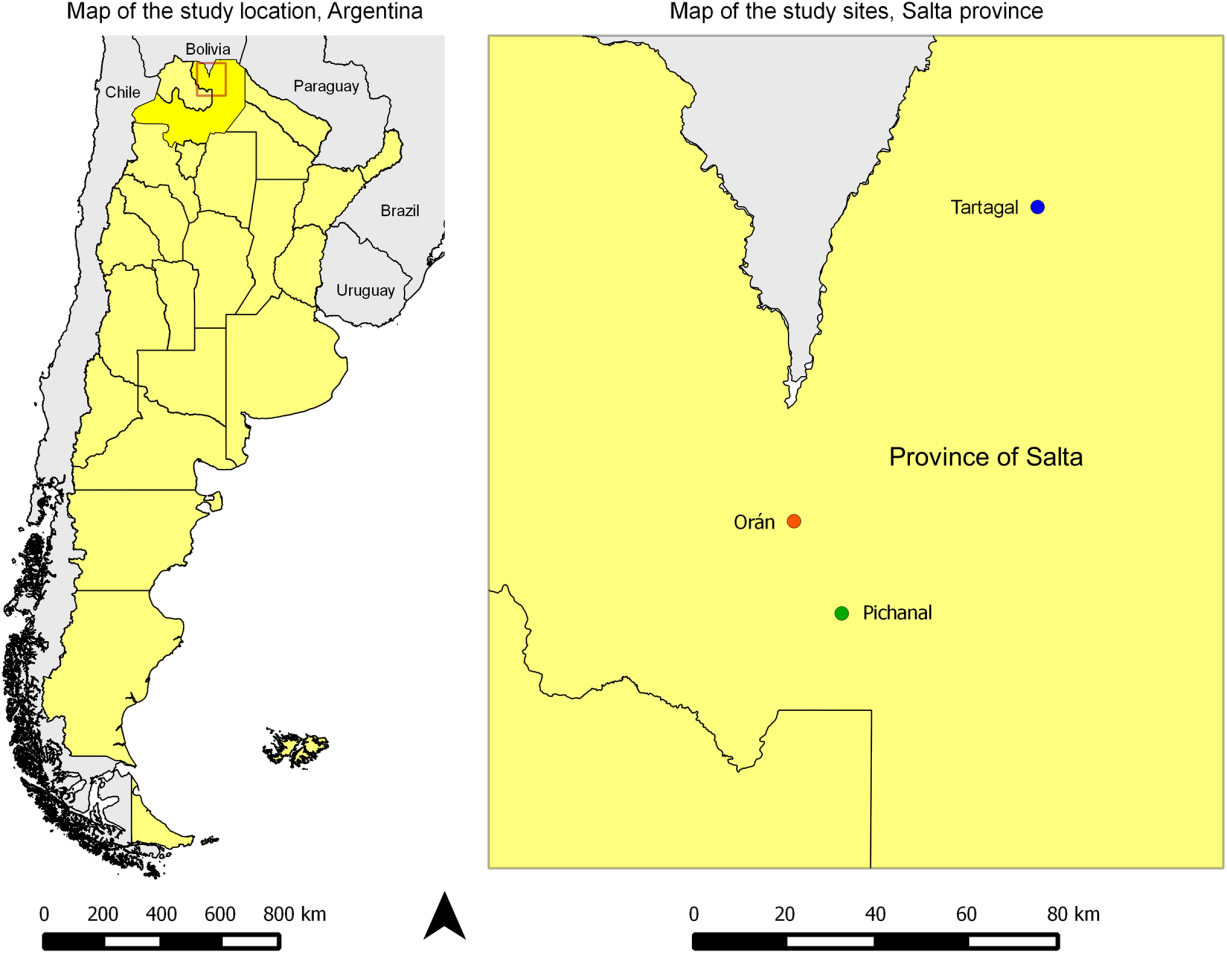
Map of the study location, Orán, Tartagal and Pichanal, Northeast of Salta province, Argentina.

This study took place in rural and urban communities from Tartagal, Pichanal and Oran; three localities placed in Salta province, Argentina, in a transition area between “Yunga” Rainforest and “Gran Chaco” regions. The climate of Salta is tropical with an annual average temperature of 22°C. (Fig 1).

All members of these communities were invited to participate. A statistically significant sample was selected for assessment through a random generated list with the household as the unit of randomization. Each inhabitant of the randomly selected houses, of any age and gender, was asked for a stool sample. Recruitment started in September 2010 and finished in March 2014.

All the subjects who provided a stool sample and consented were enrolled in the study. For the purpose of risk assessment, the subjects enrolled were classified as infected or uninfected with: 1) Skin-penetrators: based on the presence or absence of any hookworm species and/or *S*. *stercoralis* either by parasitology or serology. Two case definitions were used in the skin-penetrators group in order to discriminate the potential confounding effect of a less than optimal specificity of the NIE-ELISA serology for *S*. *stercoralis* infections a) patients with a stool examination positive for hookworm and/or *S*. *stercoralis*, and b) patients with a positive parasitological test for hookworm and/or *S*. *stercoralis* and/or a positive serological test for *S*.*stercoralis*. 2) Orally-ingested: based on the presence or absence of *T*. *trichiura* and/or *A*. *lumbricoides* eggs in the stool examination. Individuals who did not want to participate; children who did not assent or whose parents did not consent, anyone who provided an inappropriate or insufficient stool sample and subjects who received anthelminthic treatment within the previous six months were excluded from the study.

### Socio-demographic assessment

The communities included in the study are covered by a public primary health care system, with house visits by trained health personnel (“sanitary agent”) and a complete census and survey of each community every three months, collecting data about housing characteristics and demographic and sanitary data of each family member through direct observation and questionnaires. Data regarding sanitation and water supply is collected by direct observation using the classification proposed by WHO/UNICEF Joint Monitoring Program (JMP) for Water Supply and Sanitation in the sanitation and drinking-water ladders [26,27]. The household flooring material is also observed and classified into i) dirt floor (having dirt or natural floor in all the rooms of the house) and ii) concrete or tile floor (having concrete or tile as flooring material in all the rooms of the house). Data on personal hygiene behavior and practices of the study subjects is not routinely collected by the sanitary agents. Copies of the study communities’ surveys were obtained and the demographic and socioeconomic information entered in a database.

### Stool evaluation

The diagnostic approach was adapted to a population with presence of *S*. *stercoralis*. A single stool sample collected without preservatives was required from each participant. During a first surveillance-visit, sterile containers and instructions were distributed to each house and collected the following morning. Stool samples were dated, coded and then four experienced lab personnel performed the analysis, within 24 hours of collection. Five parasitological techniques were used, listed below by priority: 1) sedimentation concentration, 2) agar plate culture, 3) Harada-Mori filter paper culture, 4) Baermann concentration of charcoal-cultured fresh stool and 5) McMaster egg counting method; as described elsewhere [28,29]. If the sample volume was insufficient, concentration was the unique method performed due to its higher sensitivity for the diagnosis of *S*. *stercoralis* [30]. All the samples were read separately by the lab team manager using microscopy to identify STH eggs and larvae. All methods were grouped in a single parasitological result for each STH species, as positive if at least one reading was positive and negative if all the readings were negative.

### Serologic test for *Strongyloides stercoralis*

The individuals enrolled in the study, which agreed and signed an informed consent form, had a 5 mL blood sample drawn through venipuncture during a second surveillance-visit. All blood samples were centrifuged and an aliquot of serum was preserved frozen at –20°C and analyzed with the in-house enzyme-linked immunosorbent assay (NIE-ELISA) method for the diagnosis of *S*. *stercoralis*. NIE-ELISA detects IgG antibodies against the bacterially produced NIE recombinant antigen of *S*. *stercoralis* L3 larvae, as has been described previously [31]. Sensitivity of 75.4% and specificity of 94.8% of this method have been reported in a blinded study [32]. Patient’s sera were tested in duplicate and compared to a standard positive IgG curve based on a standard curve run on each plate. The averages of duplicate results were calculated and corrected for background reactivity (no serum added).

All the subjects whose IgG titers against NIE antigen determined by ELISA, were above the selected cutoff value were defined as seropositive for *S*. *stercoralis* and entered in a database as cases.

### Statistical analysis

Sample size was estimated using EPIDAT 3.1 (PAHO, Washington, DC); considering a predicted prevalence of 50%, a confidence level of 95%, an accuracy of 5%, and a design effect of 2; the estimated sample size was n = 730.

A description of the study population was carried out using frequencies; proportions and comparison of proportion differences between infected and uninfected subjects within each category of STH infection. Continuous variables with normal distribution were analyzed with mean, standard deviation and T-test. Continuous variables without normal distribution were analyzed with median, inter-quartile range (IQR), and Mann-Whitney test. Statistical significance was assessed by Chi-square test with 95% significance. *P* values < 0.05 were considered significant. Associations between unimproved water and sanitation and STH infection were explored through stratified bivariate analysis. Mantel-Haenszel homogeneity test was performed to control for potential confounders. Interactions between risk factors, as well as potential confusion related to sex or age, were adjusted through a multivariate logistic regression model.

The data were entered in a database designed in Microsoft Access 11.5 (Microsoft, Redmond, WA) with an Epi Info 3.5.4 (CDC, Atlanta, GA) view. Duplicate data entry was performed by three different trained collaborators. The analysis of the data was performed with the software Stata 11.0 (StataCorp LP, College Station TX); EPIDAT 3.1 (PAHO, Washington, DC) and R 3.1.1 (The R Foundation for Statistical Computing, GNU General Public License).

### Ethical statement

The research protocol and informed consent forms were evaluated and approved by the Teaching and Research Committee of the local and regional public health authorities, by the Bioethics Committee of the Colegio de Medicos de la Provincia de Salta and by the Bioethics Committee of the Faculty of Health Sciences at the Universidad Nacional de Salta. All participants provided written informed consent prior to the study. Before starting recruitment, permission was obtained from the community leaders. All the individuals living in the study communities were offered anthelmintic therapy regardless of their participation in the study.

### Deworming

This study is part of a wider project aiming to define strategies for STH control. MDA with single doses of albendazole (400 mg) and ivermectin (200 μg/kg) was applied after the study and once per year afterwards. The drugs were distributed for free, using a community house-to-house approach. The analysis of coverage, effectiveness, acceptability and feasibility of the chosen therapeutic strategy was not an objective of the present study.

## Results

6957 people were included in the census and received anthelmintics. 771 individuals were enrolled for the study. 432 of the recruited subjects provided stool and sera samples, the other 339 provided only stool samples. Missing serum samples were due to lack of consent for blood draw or absence from home at the moment of the second surveillance-visit of some of the study subjects. Table 1 summarizes the characteristics of the study population.

**Table 1:** Demographic and epidemiologic characteristics of the study population, N = 771.

Variable	N	%	95% CI
**Median age ± IQR**	16.4 ± 17	-	-
**Age group**			
Pre-school-age children †	136	17.6%	15–20
School-age children‡	379	49.1%	45–53
Adolescents and adults •	256	33.2%	30–36
**Female sex**	428	55.5%	52–59
**Urban**	271	35.1%	31–38
**Locality**			
Tartagal	316	41%	37–44
Orán	403	52%	49–56
Pichanal	52	7%	5–8
**Improved sanitation** *	232	30.1%	27–33
**Improved water supply** *	525	68.1%	65–71
**Concrete or tile floor**	362	47%	43–50
**STH infection**			
Any STH	334	43.3%	40–47
*S*. *stercoralis* infection	203	26.3%	23–29
Hookworms infection	164	21.3%	18–24
*A*. *lumbricoides* infection	63	8.2%	6–10
*T*. *Trichiura* infection	11	1.4%	0.5–2.3

†: Preschool-age children (1 to 4 years old).

‡: School-age children (5 to 14 years old).

•: Adolescents and adults (≥ 15 years old).

*Categorization of water supply and sanitation as improved or unimproved is based in the WHO/UNICEF JMP definitions [26,27].

**Fig 2:**
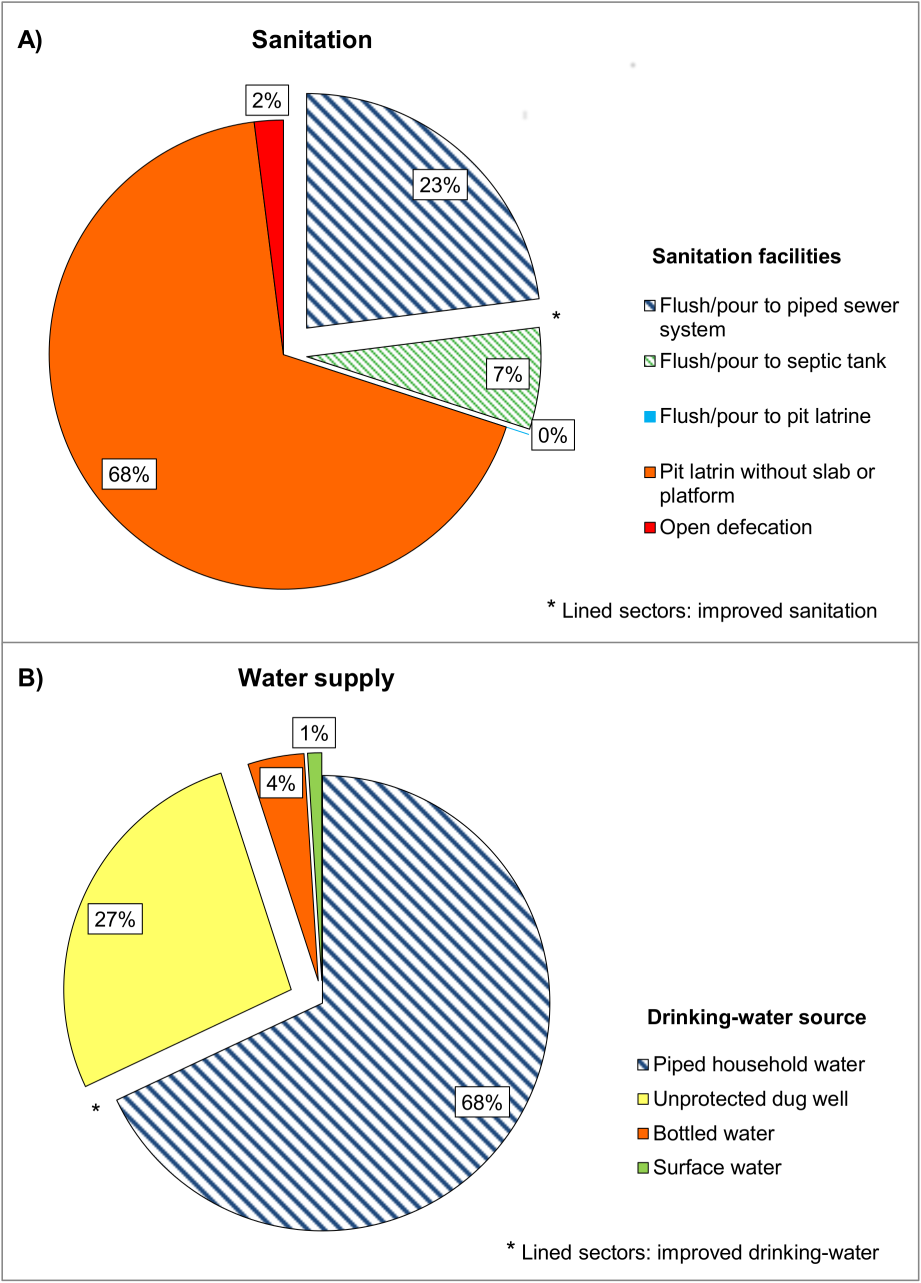
Distribution of sanitation facilities and water supply available in the study population (n = 771). A) Sanitation facilities. B) Water sources.

Improved drinking-water suply was available for 5364 (77%) individuals of the populationand improved sanitation was available for 1680 (24%) individuals. Among the assessed individuals improved water access, which consisted in piped water (previously treated for human consumption) with the connection inside the dwelling or yard, was found in 525 (68.1%) of the subjects; improved sanitation was found in 232 (30.1%) of the subjects (see Fig 2 for details).

The STH cumulative prevalence was 43.3% (n = 334; 95% CI: 40%–47%). Skin- penetrating STH were found in 37.5% (n = 289; 95% CI 34%–41%) individuals; while just 9.4% (n = 73; 95% CI: 7%–11%) of the 771 subjects assessed were infected with orally-ingested STH species. *S*. *stercoralis* was the most common species, with a prevalence of 26.3% (n = 203; 95% CI: 23%–29%); 29 of these subjects were found infected with *S*. *stercoralis* in the stool examination, but lacked NIE-ELISA serological evaluation. Among the 432 individuals who were studied by parasitology and serology, 29 were positive by both methods; 15 were positive in the stool examination and 130 were positive just by serology.

Hookworm infection followed with a prevalence of 21.3% (n = 164; 95% CI: 18%–24%). Among them, *A*. *duodenale* and *N*. *americanus* were both identified through Harada-Mori technique in 59 cases, 51 (86%; CI 95%: 77%–96%) were *A*. *duodenale* and 8 (13%; CI 95%: 4%–23%) were *N*. *americanus*. Table 2 summarizes the demographic and housing characteristics of the study population within both categories of STH infection, including the crude odds ratio of the association with risk factors.

**Table 2:** Characteristics of the study groups of soil transmitted helminths infection (n = 771).

Variable	Skin-penetrator STH species					Orally-ingested STH species				
	Infected N = 289 (%)	Uninfected N = 482 (%)	*P* value	OR*	95% CI	Infected N = 73 (%)	Uninfected N = 698 (%)	*P* value	OR*	95% CI
**Median age ±IQR**	11 ± 18	11 ± 17	0.531	1.0	0.9–1.0	10 ± 12	11 ± 18	0.417	1.0	0.9–1.0
**Female sex**	149 (61.6%)	279 (54.9%)	0.268	1.3	0.9–1.7	45 (61.6%)	383 (54.9%)	0.087	0.7	0.4–1.2
**Unimproved sanitation**	246 (85.1%)	293 (60.8%)	<0.001	3.7	2.5–5.5	44 (60.3%)	495 (70.9%)	0.059	0.6	0.4–1.1
**Unimproved water supply**	95 (32.9%)	151 (31.3%)	0.656	1.1	0. 8–1.5	33 (45.2%)	213 (30.5%)	0.010	1.9	1.1–3.1
**Dirt floor**	169 (59.7%)	233 (48.4%)	0.003	1.6	1.2–2.1	42 (58.3%)	360 (52%)	0.3699	1.3	0.0–2.2

*: Crude odds ratio reported.

We found a significant association of unimproved sanitation with any STH infection (adjusted odds ratio [aOR] = 2.4; 95% CI: 1.6–3.5) and with skin-penetrators infection (aOR = 3.9; 95% CI: 2.6–5.9). The association was significant with both case definitions, including only individuals positive by parasitology in the analysis (OR = 6.3; 95% CI: 3.7–11.2) and also including individuals positive by serology for *S*. *stercoralis* and/or parasitology (aOR = 3.9; 95% CI: 2.6–5.9). Unimproved sanitation was found to increase the odds of skin-penetrators infection regardless of the diagnostic method used for the detection of *S*. *stercoralis*, whether more sensitive or specific. Unimproved sanitation was not a risk for orally acquired STH infections (aOR = 0.4; 95% CI: 0.2–0.7).

**Fig 3:**
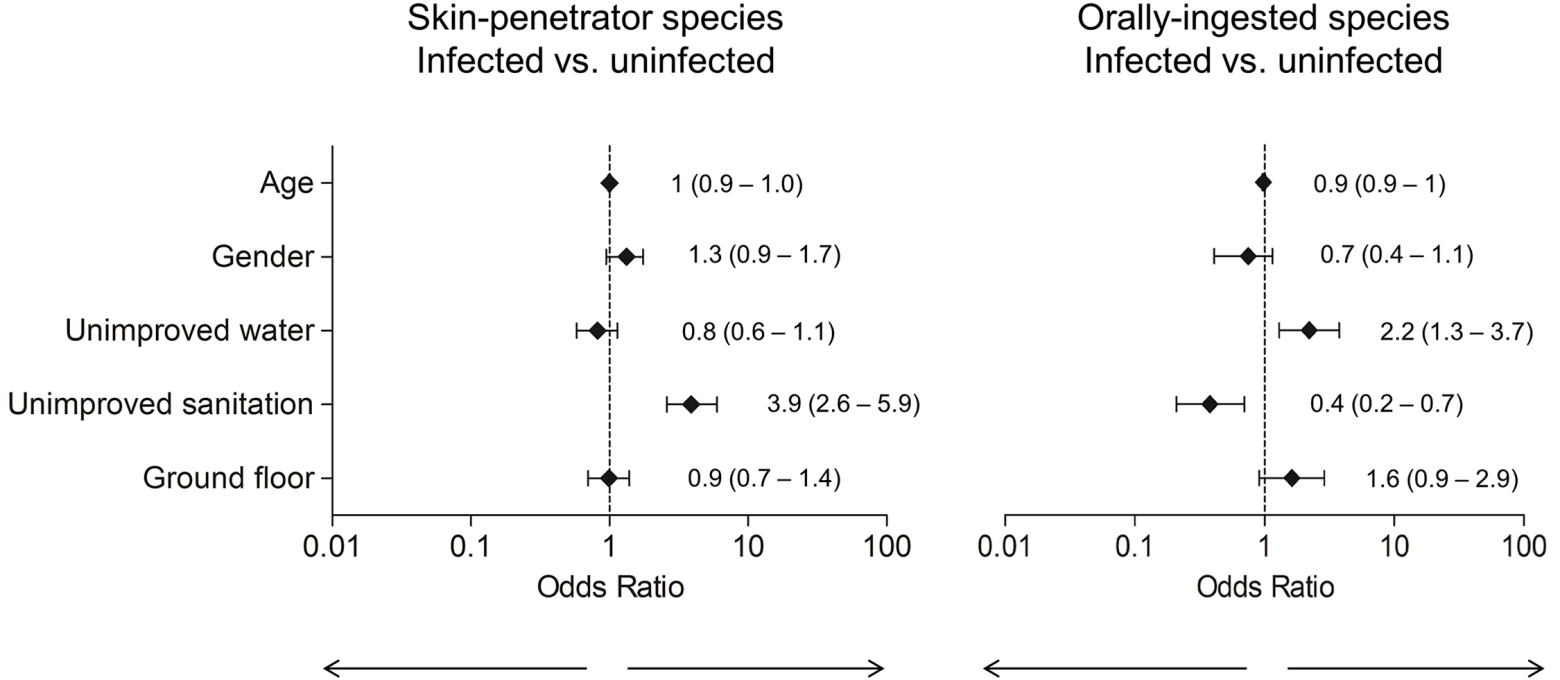
Adjusted Odds Ratios and 95% Confidence Intervals of risk factors associated with skin-penetrators and orally-ingested STH infection (n = 771)*. * The model was corrected for the possible confounders of age and sex.

No significant association between unimproved water and any STH infection was found (aOR = 0.9; 95% CI: 0.7–1.3); neither between unimproved water and skin-penetrators (aOR = 0.8; 95% CI: 0.6–1.1). We found significant association between unimproved water supply and infection with orally-ingested STH species (aOR = 2.2; 95% CI: 1.3–3.8). Fig 3 shows the adjusted ORs and 95% Confidence Intervals of uninfected vs. infected for the risk factors associated with STH infection, according to the study category.

In the species-specific analysis, unimproved sanitation was associated with *S*. *stercoralis* infection (aOR = 2.3; 95% CI: 1.5–3.6) and with hookworm infection (aOR = 7.3; 95% CI: 4–14.3). Unimproved water supply was significantly associated with *A*. *lumbricoides* infection (aOR = 2; 95% CI: 1.1–3.5) and with *T*. *trichiura* infections (aOR = 3.9; 95% CI: 1.1–19.4). Table 3 summarizes the associations found between unimproved water and sanitation and STH infection by group and species.

**Table 3:** Adjusted odds ratio (aOR) and 95% Confidence Intervals of the association between STH infections with sanitation and water access, according to mechanism of entry and species-specific (n = 771).

Infection	Unimproved sanitation access			Unimproved water access		
	aOR	95% CI	*P* value	aOR	95% CI	P value
**Any STH species**	2.4	1.6–3.5	<0.001	0.9	0.7–1.3	0.82
**Skin-penetrator species**	3.9	2.6–5.9	<0.001	0.8	0.6–1.1	0.25
•*Strongyloides stercoralis*	2.3	1.5–3.6	<0.001	1.2	0.8–1.6	0.39
•Hookworm	7.3	4.0–14.3	<0.001	0.4	0.3–0.6	<0.001
**Orally-ingested species**	0.4	0.2–0.7	0.001	2.2	1.3–3.7	0.002
•*Ascaris lumbricoides*	0.3	0.2–0.6	<0.001	2.0	1.1–3.5	0.01
•*Trichuris trichiura*	1.8	0.3–37.7	0.58	3.9	1.1–19.4	0.05

## Discussion

A massive disease burden, that includes STH among other diseases of poverty is associated with deficient access to water and sanitation [20]. The benefits of improved water supply, sanitation and hygiene outweigh their impact in health [33,34]. Many studies reported that poor access to water and sanitation are risk factors for STH infection [21,22]; however, the evidence on this association remains limited and needs further study.

The main goal of this study was to explore the relationship between water and sanitation with a key biologic and epidemiologic step in the life cycle of STH, as is the entry into the human host. Our study showed that inadequate sanitation increases the odds of infection with skin penetrating STH species and unimproved water supply increases the odds of infection with orally-ingested STH species. In the other hand, we found that neither lack of sanitation is a risk factor for orally-ingested infections, nor unimproved water supply is a risk factor for skin penetrating STH. These associations remained significant when analyzed by species (both hookworms as a single species). The finding of a seemingly protective effect of unimproved sanitation towards infections by *A*. *lumbricoides* was surprising and needs further confirmation in communities with higher prevalence of this species. The presence of dirt floor within the household, which has been described as a potential risk factor for STH infections in other studies [35], seemed to be associated with skin-penetrating STH infection but the association disappeared after adjustment.

A recent systematic review and meta-analysis by Strunz *et al*. assessed the association between WASH and STH infection [21]. They found reduced odds of STH infection related to water access and lower likelihood of infection with any STH associated with sanitation access; their findings regarding sanitation were similar to those described by Ziegelbauer *et al* [22]. The results were not as consistent regarding the association of WASH access with species specific STH burden. Sanitation access was associated with lower odds of *T*. *trichiura* and *A*. *lumbricoides* infections but no significant association was found with hookworms and the results regarding *S*. *stercoralis* were contradictory. The use of piped water reduced specifically the likelihood of *T*. *trichiura* and *A*. *lumbricoides* infections. The data on hookworm was insufficient to conduct a meta-analysis. In another meta-analysis, Esrey *et al* found an association between ascariasis and availability of drinking water and water for domestic hygiene but negligible impact of water and sanitation on hookworm infections. *T*. *trichiura* and *S*. *stercoralis* infections were not included in the analysis [20]. Our study found a statistically significant association between unimproved sanitation and hookworm infection that other studies failed to find, except for Ziegelbauer *et al*. [22] who described a protective effect provided by the availability of sanitation on hookworm infections.

Our study has limitations that require consideration for the proper interpretation of the results and the design of future steps. The sample selection process is a potential source of bias since we used the households as unit randomization even though the units of study were the individuals; an effect of design of two was used for the sample size estimation, so a larger sample was selected, to minimize this bias. Regarding diagnostics, the less than optimal sensitivity of the techniques, particularly for low intensity infections, which is most significant for the case of *S*. *stercoralis* [5], is a limitation of the diagnostic approach that has been in part controlled by the use of several techniques. In order to evaluate the effect of more sensitive tools, an alternative case definition using the NIE-ELISA serology as added criteria for cases of *S*. *stercoralis* was explored, with the associations remaining significant; furthermore, residual antibody titers after cure are unlikely with the NIE-ELISA as has been shown that titers fall after a few months after cure [36].

Our study does not report results regarding the association of risk factors with egg burden because the McMaster method was not prioritized, resulting in fewer samples analyzed with this quantitative method. An additional source of potential underestimation of prevalence, particularly for hookworms, was the time spent between sample collection and analysis that was due to the remoteness of some of the study sites from the laboratory, which varied from 6 to 110 kilometers [37]. Another limitation of our study is that we registered availability and type of sanitation and water facilities at the household level without any inference on the use, quality and maintenance of these facilities. We neither obtained any information regarding availability and characteristics of sanitation facilities at the local schools and working environments. Hygiene was not considered in our assessment because data on hygiene practices were not collected in the socio demographic survey. Therefore, our results are an approximation to the issue of the effect of sanitation and water on STH infection that needs to be further studied. However, the strength of the associations found and the consistency between water and sanitation availability and the STH prevalence found supports the hypothesis of selectiveness of the effect of unimproved sanitation and water on the infective mechanism.

Our findings have direct implications on current strategic plans for the control and elimination of STH and may contribute to improve the current recommendations for the control of STH. The current assumption that the same control measures are useful for all the different STH could be challenged by these findings. Knowledge on the characteristics of water and sanitation access in a given community might help in the selection of the anthelminthic of choice for MDA programs in view of the variable activity of the drugs against different STH species [38]. By the same token, surveys that identify certain patterns of species distribution could be used in the advocacy for water and/or sanitation improvements from policy makers. Renewed efforts for modeling and mathematical estimations of deworming needs for the elimination of STH [39,40], could incorporate these categorization of STH infection based in the mechanism of entry in future studies in order to describe different scenarios for intervention.

In summary, our analysis shows the value of evaluating the impact of water and sanitation using the house availability of each of these variables, through the segregation of STH species based on the route of entry in a phenomenological epidemiologic approach without implication to the details of the mechanisms involved.

## Supporting Information

S1 Checklist STROBE statement for observational studies checklist. (PDF)

S1 Text Readme file of the database: *Dataset_Argentina_STH_WS_771*. (TXT)

S2 Text Readme file of the database: *Dataset_Argentina_STH_WS_6957*. (TXT)

S1 Dataset Socio-demographic data and results of the diagnostic methods performed of the 771 subjects enrolled in the study. (CSV)

S2 Dataset Socio-demographic data of the full population of the study communities (n = 6957). (CSV)

S1 Table Table summarizing socio-demographic characteristics of the full population of the study communities (n = 6957). (DOCX)

## References

[1] De Silva NR, Brooker S, Hotez PJ, Montresor A, Engels D, Savioli L. Soil-transmitted helminth infections: Updating the global picture. Trends Parasitol. 2003;19(12):547–51. 14642761 10.1016/j.pt.2003.10.002

[2] Brooker S, Clements a. C a, Bundy D a P. Global Epidemiology, Ecology and Control of Soil-Transmitted Helminth Infections. Adv Parasitol. 2006;62(05):221–61. 16647972 10.1016/S0065-308X(05)62007-6 PMC1976253

[3] Bethony J, Brooker S, Albonico M, Geiger SM, Loukas A, Diemert D, et al Soil-transmitted helminth infections: ascariasis, trichuriasis, and hookworm. Lancet. 2006;367 16679166 10.1016/S0140-6736(06)68653-4

[4] Olsen A, van Lieshout L, Marti H, Polderman T, Polman K, Steinmann P, et al Strongyloidiasis—the most neglected of the neglected tropical diseases? Trans R Soc Trop Med Hyg. 2009;103(10):967–72. 10.1016/j.trstmh.2009.02.013 19328508

[5] Krolewiecki AJ, Lammie P, Jacobson J, Gabrielli AF, Levecke B, Socias E, et al A Public Health Response against Strongyloides stercoralis: Time to Look at Soil-Transmitted Helminthiasis in Full. PLoS Negl Trop Dis. 2013;7(5):1–7. 10.1371/journal.pntd.0002165 PMC3649958 23675541

[6] Crompton DWT, Nesheim MC. Nutritional impact of intestinal helminthiasis during the human life cycle. Annu Rev Nutr. 2002;22:35–59. 12055337 10.1146/annurev.nutr.22.120501.134539

[7] Hotez P, Molyneux D, Fenwick A, Kumaresan J, Ehrlich Sachs S, Sachs J, et al Control of neglected tropical diseases. N Engl J Med. 2007;1018–27. 17804846 10.1056/NEJMra064142

[8] Albonico M, Allen H, Chitsulo L, Engels D, Gabrielli AF, Savioli L. Controlling soil-transmitted helminthiasis in pre-school-age children through preventive chemotherapy. PLoS Negl Trop Dis. 2008;2(3). 10.1371/journal.pntd.0000126 PMC2274864 18365031

[9] World Health Organization Working to overcome the global impact of neglected tropical diseases First WHO report on neglected tropical diseases Crompton DWT, editor. France: WHO Press; 2010.

[10] Taranto NJ, Cajal SP, De Marzi MC, Fernández MM, Frank FM, Brú a M, et al Clinical status and parasitic infection in a Wichi Aboriginal community in Salta,Argentina. Trans R Soc Trop Med Hyg. 2003;97(5):554–8. 15307425 10.1016/s0035-9203(03)80026-3

[11] Socías M, Fernandez A, Gil J, Krolewiecki A. Goehelmintiasis en la Argentina Una Revisión Sistemática. Med (Buenos Aires). 2014;74:29–36. 24561837

[12] Instituto Nacional de Estadística y Censos (INDEC). Censo Nacional de Población, Hogares y Viviendas 2010 Censo del Bicentenario. Resultados definitivos serie B Nro 2. 2012.

[13] Dirección Nacional de Relaciones Económicas con las Provincias (DINREP). Necesidades Básicas Insatisfechas (NBI). Información Censal del año 2010. Buenos Aires: Ministerio de Economía y Finanzas Públicas de la Nación Argentina; 2014. p. 53–4.

[14] World Health Organization. Preventive Chemotherapy in Human Helminthiasis: Coordinated Use of Anthelminthic Drugs in Control Interventions-A Manual for Health Professionals. 2006;

[15] World Health Organization. Helminth control in school-age children. A guide for managers of control programs. Second edition. 2011;

[16] Gass K, Addiss DG, Freeman MC. Exploring the Relationship between Access to Water, Sanitation and Hygiene and Soil-Transmitted Helminth Infection: A Demonstration of Two Recursive Partitioning Tools. PLoS Negl Trop Dis. 2014;8(6). 10.1371/journal.pntd.0002945 24921253 PMC4055441

[17] Jia T-W, Melville S, Utzinger J, King CH, Zhou X-N. Soil-Transmitted Helminth Reinfection after Drug Treatment: A Systematic Review and Meta-Analysis. PLoS Negl Trop Dis. 2012;6(5):e1621 10.1371/journal.pntd.0001621 22590656 PMC3348161

[18] Campbell SJ, Savage GB, Gray DJ, Atkinson JM, Soares RJ, Nery S V, et al Water, Sanitation, and Hygiene (WASH): A Critical Component for Sustainable Soil-Transmitted Helminth and Schistosomiasis Control. 2014;8(4):1–5. 10.1371/journal.pntd.0002651 PMC3983087 24722335

[19] Hawdon JM. Controlling soil-transmitted helminths: time to think inside the box? J Parasitol. 2014;100(2):166–88. 10.1645/13-412.1 24393037

[20] Esrey S a, Potash JB, Roberts L, Shiff C. Reviews / Analyses Effects of improved water supply and sanitation on. Bull World Health Organ. 1991;69(5):609–21. 1835675 PMC2393264

[21] Strunz EC, Addiss DG, Stocks ME, Ogden S, Freeman MC. Water, Sanitation, Hygiene, and Soil-Transmitted Helminth Infection: A Systematic Review and Meta-Analysis. PLoS Med. 2014;11(3). 10.1371/journal.pmed.1001620 24667810 PMC3965411

[22] Ziegelbauer K, Speich B, Mäusezahl D, Bos R, Keiser J, Utzinger J. Effect of sanitation on soil-transmitted helminth infection: Systematic review and meta-analysis. PLoS Med. 2012;9(1). 10.1371/journal.pmed.1001162 22291577 PMC3265535

[23] Asaolu SO, Ofoezie IE. The role of health education and sanitation in the control of helminth infections. Acta Trop. 2003;86(2–3):283–94. 12745145 10.1016/s0001-706x(03)00060-3

[24] Bundy D. Is the hookworm just another geohelminth? In: Schad G, Warren K, editors. Hookworm disease current status and new directions. London: Taylor and Francis; 1990 p. 147–64.

[25] Zeehaida M, Zairi NZ, Rahmah N, Maimunah A, Madihah B. Strongyloides stercoralis in common vegetables and herbs in Kota Bharu, Kelantan, Malaysia. Trop Biomed. 2011;28(1):188–93. 21602786

[26] World Health Organization and UNICEF. Joint Monitoring Programme for Water Supply and Sanitation. Core questions on drinking-water and sanitation for household surveys. WHO/UNICEF Joint Monitoring Programme for Water Supply and Sanitation. WHO Press, World Health Organization, 20 Avenue Appia, 1211 Geneva 27, Switzerland; 2006.

[27] WHO/UNICEF Joint Monitoring Programme for Water Supply and Sanitation. Progress on Sanitation and Drinking-water: 2010 Update Switzerland: WHO Press, World Health Organization; 2010.

[28] Garcia L. Diagnostic medical parasitology 4th ed. Diagnostic. Washington DC: ASM press; 2001.

[29] Levecke B, Behnke JM, Ajjampur SSR, Albonico M, Ame SM, Charlier J, et al A comparison of the sensitivity and fecal egg counts of the McMaster egg counting and Kato-Katz thick smear methods for soil-transmitted helminths. PLoS Negl Trop Dis. 2011 6;5(6):e1201 10.1371/journal.pntd.0001201 21695104 PMC3114752

[30] Krolewiecki AJ, Ramanathan R, Fink V, McAuliffe I, Cajal SP, Won K, et al Improved Diagnosis of Strongyloides stercoralis Using Recombinant Antigen-Based Serologies in a Community-Wide Study in Northern Argentina. Clin Vaccine Immunol. 2010 8;17(10):1624–30. 10.1128/CVI.00259-10 20739501 PMC2952987

[31] Ravi V, Ramachandran S, Thompson RW, Andersen JF, Neva F a. Characterization of a recombinant immunodiagnostic antigen (NIE) from Strongyloides stercoralis L3-stage larvae. Mol Biochem Parasitol. 2002;125(1–2):73–81. 12467975 10.1016/s0166-6851(02)00214-1

[32] Bisoffi Z, Buonfrate D, Sequi M, Mejia R, Cimino RO, Krolewiecki AJ, et al Diagnostic accuracy of five serologic tests for Strongyloides stercoralis infection. PLoS Negl Trop Dis. 2014 1;8(1):e2640 10.1371/journal.pntd.0002640 24427320 PMC3890421

[33] Bartram J, Cairncross S. Hygiene, sanitation, and water: Forgotten foundations of health. PLoS Med. 2010;7(11):1–9. 10.1371/journal.pmed.1000367 PMC2976722 21085694

[34] Albonico M, Montresor A, Crompton DWT, Savioli L. Intervention for the Control of Soil-Transmitted Helminthiasis in the Community. Adv Parasitol. 2006;61(05):311–48. 16735168 10.1016/S0065-308X(05)61008-1 PMC5633078

[35] Liu C, Luo R, Yi H, Zhang L, Li S, Bai Y, et al Soil-Transmitted Helminths in Southwestern China: A Cross-Sectional Study of Links to Cognitive Ability, Nutrition, and School Performance among Children. PLoS Negl Trop Dis. 2015 6;9(6):e0003877 10.1371/journal.pntd.0003877 26110518 PMC4481344

[36] Buonfrate D, Sequi M, Mejia R, Cimino RO, Krolewiecki AJ, Albonico M, et al Accuracy of five serologic tests for the follow up of Strongyloides stercoralis infection. PLoS Negl Trop Dis. 2015 2;9(2):e0003491 10.1371/journal.pntd.0003491 25668740 PMC4323101

[37] Dacombe RJ, Crampin a C, Floyd S, Randall A, Ndhlovu R, Bickle Q, et al Time delays between patient and laboratory selectively affect accuracy of helminth diagnosis. Trans R Soc Trop Med Hyg. 2007 2;101(2):140–5. 16824566 10.1016/j.trstmh.2006.04.008

[38] Keiser J, Utzinger J. Efficacy of current drugs against soil-transmitted helminth infections. Systematic review and meta-analysis. JAMA. 2008;299(16):1937–48. 10.1001/jama.299.16.1937 18430913

[39] Anderson RM, Truscott JE, Hollingsworth TD. The coverage and frequency of mass drug administration required to eliminate persistent transmission of soil-transmitted helminths. Philos Trans R Soc Lond B Biol Sci. 2014;369(1645):20130435 10.1098/rstb.2013.0435 24821921 PMC4024228

[40] Truscott JE, Hollingsworth TD, Brooker SJ, Anderson RM. Can chemotherapy alone eliminate the transmission of soil transmitted helminths? Parasit Vectors. 2014;7(1):266. 24916278 10.1186/1756-3305-7-266 PMC4079919

