# Supplementary material for: Effect of Poor Access to Water and Sanitation As Risk Factors for Soil-Transmitted Helminth Infection: Selectiveness by the Infective Route
Source: PLoS Negl Trop Dis. 2015 Sep 30;9(9):e0004111. doi: 10.1371/journal.pntd.0004111 (PMC4589369; doi:10.1371/journal.pntd.0004111)
Supplement: S1 Checklist — (PDF) [file pntd.0004111.s001.pdf]

STROBE Statement—checklist of items that should be included in reports of observational studies

	Item No	Recommendation	Page No (line)	Relevant text from manuscript
**Title and abstract**	1	(*a*) Indicate the study's design with a commonly used term in the title or the abstract	2 (31)	Cross-sectional study, conducted in Salta province, Argentina.
		(*b*) Provide in the abstract an informative and balanced summary of what was done and what was found	2 – 3 (31-41)	771 were randomly selected for stool/serum sampling for parasitological and serological diagnosis of STH. Bivariate stratified analysis was performed to explore significant correlations between risk factors and STH infections grouped by mechanism of entry as skin-penetrators (hookworms and *Strongyloides stercoralis*) vs. orally-ingested (*Ascaris lumbricoides* and *Trichuris trichiura*)… unimproved sanitation was significantly associated with increased odds of infection of skin-penetrators (adjusted odds ratio [aOR]=3.9; 95% CI: 2.6–5.9). Unimproved drinking water was significantly associated with increased odds of infection of orally-ingested (aOR=2.2; 95% CI: 1.3–3.7).
**Introduction**				
Background /rationale	2	Explain the scientific background and rationale for the investigation being reported	5 (85-87) 6 (100-103)	WASH interventions represent a strategy to preserve the benefit of chemotherapy by preventing the uninfected to be exposed, which is sustainable and can eventually lead to transmission interruption and STH eradication (19–22).<br>Based on their mechanism of entry, STH species can be classified into two groups i) skin-penetrators and ii) orally-ingested. This distinction might be relevant to find specific associations between a risk factor and an infective route.
Objectives	3	State specific objectives, including any prespecified hypotheses	6 (103-107)	We hypothesized that lacking access to water and sanitation increases the risk of infection with specific STH species, depending on their mechanism of entry. We conducted a cross-sectional study to examine the

				independent effect of inadequate water supply and sanitation, on the prevalence of STH species grouped by mechanism of entry (skin-penetrators vs. orally-ingested).
**Methods**				
Study design	4	Present key elements of study design early in the paper	7 (112-114)	*Study design*<br>Population based cross-sectional study, conducted in the localities of Tartagal, Orán and Pichanal of Salta Province, Argentina.
Setting	5	Describe the setting, locations, and relevant dates, including periods of recruitment, exposure, follow-up, and data collection	7 (115-119)<br>7 (126)	*Study population*<br>This study took place in rural and urban communities from Tartagal, Pichanal and Oran; three localities placed in Salta province, Argentina, in a transition area between "Yunga" Rainforest and "Gran Chaco" regions. The climate of Salta is tropical with an annual average temperature of 22°C.<br>Recruitment started in September 2010 and finished in March 2014.
Participants	6	(*a*) *Cohort study*—Give the eligibility criteria, and the sources and methods of selection of participants. Describe methods of follow-up<br>*Case-control study*—Give the eligibility criteria, and the sources and methods of case ascertainment and control selection. Give the rationale for the choice of cases and controls<br>*Cross-sectional study*—Give the eligibility criteria, and the sources and methods of selection of participants	7 (122-128)	All members of these communities were invited to participate. A statistically significant sample was selected for assessment through a random generated list with the household as the unit of randomization. Each inhabitant of the randomly selected houses, of any age and gender, was asked for a stool sample.<br>All the subjects who provided a stool sample and consented were enrolled in the study.
Variables	7	Clearly define all outcomes, exposures, predictors, potential confounders, and effect modifiers. Give diagnostic criteria, if applicable	7 - 8 (128-137)<br>10 – 11 (201-202)<br>11 (203-205)	For the purpose of risk assessment, the subjects enrolled were classified as infected or uninfected with: 1) Skin-penetrators: based on the presence or absence of any hookworm species and/or *S. stercoralis* either by parasitology or serology…2) Orally-ingested: based on the presence or absence of *T. trichiura* and/or *A. lumbricoides* eggs in the stool examination.<br>Associations between unimproved water and sanitation and STH

				infection were explored through stratified bivariate analysis. Interactions between risk factors, as well as potential confusion related to sex or age, were adjusted through a multivariate logistic regression model.
Data sources/ measurement	8*	For each variable of interest, give sources of data and details of methods of assessment (measurement). Describe comparability of assessment methods if there is more than one group	8 (147-153)<br>9 (158-173)<br>9 – 10 (174-189)	*Socio-demographic assessment*<br>Data regarding sanitation and water supply is collected by direct observation using the classification proposed by WHO/UNICEF Joint Monitoring Program (JMP) for Water Supply and Sanitation in the sanitation and drinking-water ladders (26,27). The household flooring material is also observed and classified into i) dirt floor (having dirt or natural floor in all the rooms of the house) and ii) concrete or tile floor (having concrete or tile as flooring material in all the rooms of the house).<br>*Stool evaluation*<br>A single stool sample collected without preservatives was required from each participant. During a first surveillance-visit, sterile containers and instructions were distributed to each house and collected the following morning. Stool samples were dated, coded and then four experienced lab personnel performed the analysis, within 24 hours of collection. Five parasitological techniques were used, listed below by priority: 1) sedimentation concentration, 2) agar plate culture, 3) Harada-Mori filter paper culture, 4) Baermann concentration of charcoal-cultured fresh stool and 5) McMaster egg counting method; as described elsewhere (28,29)… All the samples were read separately by the lab team manager using microscopy to identify STH eggs and larvae. All methods were grouped in a single parasitological result for each STH species, as positive if at least one reading was positive and

				negative if all the readings were negative. *Serologic test for Strongyloides stercoralis* The individuals enrolled in the study, which agreed and signed an informed consent form, had a 5 mL blood sample drawn through venipuncture during a second surveillance-visit. All blood samples were centrifuged and an aliquot of serum was preserved frozen at –20˚C and analyzed with the in-house enzyme-linked immunosorbent assay (NIE-ELISA) method for the diagnosis of *S. stercoralis*. NIE-ELISA detects IgG antibodies against the bacterially produced NIE recombinant antigen of *S. stercoralis* L3 larvae, as has been described previously (31).
Bias	9	Describe any efforts to address potential sources of bias	7 (122-124)<br>8 (143-147)<br>9 (164)<br>10 (183-186)<br>11 (207-208)	A statistically significant sample was selected for assessment through a random generated list with the household as the unit of randomization.<br>The communities included in the study are covered by a public primary health care system, with house visits by trained health personnel (“sanitary agent”) and a complete census and survey of each community every three months, collecting data about housing characteristics and demographic and sanitary data of each family member through direct observation and questionnaires<br>Five parasitological techniques were used…<br>Patient’s sera were tested in duplicate and compared to a standard positive IgG curve based on a standard curve run on each plate. The averages of duplicate results were calculated and corrected for background reactivity (no serum added).<br>Duplicate data entry was performed by three different trained collaborators.

Study size	10	Explain how the study size was arrived at	10 (191-193)	Sample size was estimated using EPIDAT 3.1 (PAHO, Washington, DC); considering a predicted prevalence of 50%, a confidence level of 95%, an accuracy of 5%, and a design effect of 2; the estimated sample size was n=730.
Quantitative variables	11	Explain how quantitative variables were handled in the analyses. If applicable, describe which groupings were chosen and why	10 (196-199)	Continuous variables with normal distribution were analyzed with mean, standard deviation and T-test. Continuous variables without normal distribution were analyzed with median, inter-quartile range (IQR), and Mann-Whitney test.
Statistical methods	12	(*a*) Describe all statistical methods, including those used to control for confounding	10 (194-196) 10 – 11 (199-205)	A description of the study population was carried out using frequencies; proportions and comparison of proportion differences between infected and uninfected subjects within each category of STH infection. Statistical significance was assessed by Chi-square test with 95% significance. *P* values < 0.05 were considered significant. Associations between unimproved water and sanitation and STH infection were explored through stratified bivariate analysis. Mantel-Haenszel homogeneity test was performed to control for potential confounders. Interactions between risk factors, as well as potential confusion related to sex or age, were adjusted through a multivariate logistic regression model.
		(*b*) Describe any methods used to examine subgroups and interactions	10 (201-202)	Associations between unimproved water and sanitation and STH infection were explored through stratified bivariate analysis.
		(*c*) Explain how missing data were addressed		
		(*d*) *Cohort study*—If applicable, explain how loss to follow-up was addressed *Case-control study*—If applicable, explain how matching of cases and controls was addressed *Cross-sectional study*—If applicable, describe analytical methods taking account of sampling strategy	10 (191-193)	Sample size was estimated using EPIDAT 3.1 (PAHO, Washington, DC); considering a predicted prevalence of 50%, a confidence level of 95%, an accuracy of 5%, and a design effect of 2; the estimated sample size was n=730.

		(*e*) Describe any sensitivity analyses		
**Results**				
Participants	13*	(a) Report numbers of individuals at each stage of study—eg numbers potentially eligible, examined for eligibility, confirmed eligible, included in the study, completing follow-up, and analysed	12 (229-231)	6957 people were included in the census and received anthelmintics. 771 individuals were enrolled for the study. 432 of the recruited subjects provided stool and sera samples, the other 339 provided only stool samples.
		(b) Give reasons for non-participation at each stage	12 (231-233)	Missing serum samples were due to lack of consent for blood draw or absence from home at the moment of the second surveillance-visit of some of the study subjects.
		(c) Consider use of a flow diagram		
Descriptive data	14*	(a) Give characteristics of study participants (eg demographic, clinical, social) and information on exposures and potential confounders	12 (235-236)<br>13 (241-246)	Table 1: Demographic and epidemiologic characteristics of the study population, N=771.<br>Improved drinking-water suply was available for 5364 (77%) individuals of the population and improved sanitation was available for 1680 (24%) individuals. Among the assessed individuals improved water access, which consisted in piped water (previously treated for human consumption) with the connection inside the dwelling or yard, was found in 525 (68.1%) of the subjects; improved sanitation was found in 232 (30.1%) of the subjects
		(b) Indicate number of participants with missing data for each variable of interest		
		(c) *Cohort study*—Summarise follow-up time (eg, average and total amount)		
Outcome data	15*	*Cohort study*—Report numbers of outcome events or summary measures over time		
		Case-control study—Report numbers in each exposure category, or summary measures of exposure		
		Cross-sectional study—Report numbers of outcome events or summary measures	12 (235-236)<br>13 – 14 (250-262)<br>14 (266-267)	Table 1: Demographic and epidemiologic characteristics of the study population, N=771.<br>The STH cumulative prevalence was 43.3% (n=334; 95% CI: 40%–47%). Skin- penetrating STH were found in 37.5% (n= 289; 95% CI 34%–41%) individuals; while just 9.4% (n=73; 95% CI: 7%–11%) of the 771 subjects assessed were infected with orally-

				ingested STH species. *S. stercoralis* was the most common species, with a prevalence of 26.3% (n=203; 95% CI: 23%–29%); 29 of these subjects were found infected with *S. stercoralis* in the stool examination, but lacked NIE-ELISA serological evaluation. Among the 432 individuals who were studied by parasitology and serology, 29 were positive by both methods; 15 were positive in the stool examination and 130 were positive just by serology. Hookworm infection followed with a prevalence of 21.3% (n= 164; 95% CI: 18%–24%). Among them, *A. duodenale* and *N. americanus* were both identified through Harada-Mori technique in 59 cases, 51 (86 %; CI 95%: 77%–96%) were *A. duodenale* and 8 (13 %; CI 95%: 4%–23%) were *N. americanus*. Table 2: Characteristics of the study groups of soil transmitted helminths infection. (n=771).
Main results	16	(*a*) Give unadjusted estimates and, if applicable, confounder-adjusted estimates and their precision (eg, 95% confidence interval). Make clear which confounders were adjusted for and why they were included	14 (262-265)<br>15 (286-288)<br>16 (296-298)	Table 2 summarizes the demographic and housing characteristics of the study population within both categories of STH infection, including the crude odds ratio of the association with risk factors. Fig 3. Adjusted Odds Ratios and 95% Confidence Intervals of risk factors associated with skin-penetrators and orally-ingested STH infection (n= 771)*. * The model was corrected for the possible confounders of age and sex. Table 3 – Adjusted odds ratio (aOR) and 95% Confidence Intervals of the association between STH infections with sanitation and water access, according to mechanism of entry and species-specific (n=771).
		(*b*) Report category boundaries when continuous variables were categorized	13 (237-238)	†: Preschool-age children (1 to 4 years old). ‡: School-age children (5 to 14 years old). •: Adolescents and adults (≥ 15 years old).
		(*c*) If relevant, consider translating estimates of relative risk into absolute risk for a meaningful time period		
Other analyses	17	Report other analyses done—eg analyses of subgroups and interactions, and sensitivity analyses	15 (289-293)	In the species-specific analysis, unimproved sanitation was associated with *S. stercoralis* infection (aOR=2.3;

				95% CI: 1.5–3.6) and with hookworm infection (aOR=7.3; 95% CI: 4–14.3). Unimproved water supply was significantly associated with *A. lumbricoides* infection (aOR=2; 95% CI: 1.1–3.5) and with *T. trichiura* infections (aOR= 3.9; 95% CI: 1.1-19.4).
**Discussion**				
Key results	18	Summarise key results with reference to study objectives	16 (306-310)	The main goal of this study was to explore the relationship between water and sanitation with a key biologic and epidemiologic step in the life cycle of STH, as is the entry into the human host. Our study showed that inadequate sanitation increases the odds of infection with skin penetrating STH species and unimproved water supply increases the odds of infection with orally-ingested STH species.
Limitations	19	Discuss limitations of the study, taking into account sources of potential bias or imprecision. Discuss both direction and magnitude of any potential bias	18 – 19 (339-370)	Our study has limitations that require consideration for the proper interpretation of the results and the design of future steps. The sample selection process is a potential source of bias since we used the households as unit randomization even though the units of study were the individuals; an effect of design of two was used for the sample size estimation, so a larger sample was selected, to minimize this bias.<br>Regarding diagnostics, the less than optimal sensitivity of the techniques, particularly for low intensity infections, which is most significant for the case of *S. stercoralis* (5), is a limitation of the diagnostic approach that has been in part controlled by the use of several techniques. In order to evaluate the effect of more sensitive tools, an alternative case definition using the NIE-ELISA serology as added criteria for cases of *S. stercoralis* was explored, with the associations remaining significant; furthermore, residual antibody titers after cure are unlikely with the NIE-ELISA as has been shown that titers fall after a few months after cure (36).<br>Our study does not report results regarding the association of risk factors with egg burden because the McMaster

				method was not prioritized, resulting in fewer samples analyzed with this quantitative method. An additional source of potential underestimation of prevalence, particularly for hookworms, was the time spent between sample collection and analysis that was due to the remoteness of some of the study sites from the laboratory, which varied from 6 to 110 kilometers (37). Another limitation of our study is that we registered availability and type of sanitation and water facilities at the household level without any inference on the use, quality and maintenance of these facilities. We neither obtained any information regarding availability and characteristics of sanitation facilities at the local schools and working environments. Hygiene was not considered in our assessment because data on hygiene practices were not collected in the socio demographic survey. Therefore, our results are an approximation to the issue of the effect of sanitation and water on STH infection that needs to be further studied. However, the strength of the associations found and the consistency between water and sanitation availability and the STH prevalence found supports the hypothesis of selectiveness of the effect of unimproved sanitation and water on the infective mechanism.
Interpretation	20	Give a cautious overall interpretation of results considering objectives, limitations, multiplicity of analyses, results from similar studies, and other relevant evidence	17 – 18 (321-338)	A recent systematic review and meta-analysis by Strunz *et al.* assessed the association between WASH and STH infection (21). They found reduced odds of STH infection related to water access and lower likelihood of infection with any STH associated with sanitation access; their findings regarding sanitation were similar to those described by Ziegelbauer *et al* (22). The results were not as consistent regarding the association of WASH access with species specific STH burden. Sanitation access was associated with lower odds of *T. trichiura* and *A. lumbricoides*

				infections but no significant association was found with hookworms and the results regarding *S. stercoralis* were contradictory. The use of piped water reduced specifically the likelihood of *T. trichiura* and *A. lumbricoides* infections. The data on hookworm was insufficient to conduct a meta-analysis. In another meta-analysis, Esrey *et al* found an association between ascariasis and availability of drinking water and water for domestic hygiene but negligible impact of water and sanitation on hookworm infections. *T. trichiura* and *S. stercoralis* infections were not included in the analysis (20). Our study found a statistically significant association between unimproved sanitation and hookworm infection that other studies failed to find, except for Ziegelbauer *et al.* (22) who described a protective effect provided by the availability of sanitation on hookworm infections.
Generalisability	21	Discuss the generalisability (external validity) of the study results	19 (371-383)	Our findings have direct implications on current strategic plans for the control and elimination of STH and may contribute to improve the current recommendations for the control of STH. The current assumption that the same control measures are useful for all the different STH could be challenged by these findings. Knowledge on the characteristics of water and sanitation access in a given community might help in the selection of the anthelminthic of choice for MDA programs in view of the variable activity of the drugs against different STH species (38). By the same token, surveys that identify certain patterns of species distribution could be used in the advocacy for water and/or sanitation improvements from policy makers. Renewed efforts for modeling and mathematical estimations of deworming needs for the elimination of STH (39,40), could incorporate these categorization of STH infection based in the mechanism of entry in future studies in order to describe different scenarios

			for intervention.
**Other information**			
Funding	22	Give the source of funding and the role of the funders for the present study and, if applicable, for the original study on which the present article is based	

*Give information separately for cases and controls in case-control studies and, if applicable, for exposed and unexposed groups in cohort and cross-sectional studies.

Note: An Explanation and Elaboration article discusses each checklist item and gives methodological background and published examples of transparent reporting. The STROBE checklist is best used in conjunction with this article (freely available on the Web sites of PLoS Medicine at http://www.plosmedicine.org/, Annals of Internal Medicine at http://www.annals.org/, and Epidemiology at http://www.epidem.com/). Information on the STROBE Initiative is available at www.strobe-statement.org.
