# Supplementary material for: Effect of Poor Access to Water and Sanitation As Risk Factors for Soil-Transmitted Helminth Infection: Selectiveness by the Infective Route
Source: PLoS Negl Trop Dis. 2015 Sep 30;9(9):e0004111. doi: 10.1371/journal.pntd.0004111 (PMC4589369; doi:10.1371/journal.pntd.0004111)
Supplement: S1 Table — (DOCX) [file pntd.0004111.s006.docx]

Additional table to Table 1: Characteristics of the full study population.

| **Characteristic** | | **N=6957 (100%)** |
| --- | --- | --- |
| **Age** | Median age ± IQR  PSAC  SAC  Adolescents and adults  Without data | 18 ± 24 |
|  |  | 1035 (15%) |
|  |  | 1936 (28%) |
|  |  | 3933 (56%) |
|  |  | 53 (0.8%) |
| **Gender** | Male | 3520 (51%) |
|  | Female | 3372 (48%) |
|  | Without data | 65 (0.8%) |
| **Environment** | Urban | 2165 (31%) |
|  | Rural | 4792 (69%) |
| **Locality** | Tartagal | 2927 (42%) |
|  | Orán | 3603 (52%) |
|  | Pichanal | 427 (6%) |
| **Sanitation*** | Improved | 1680 (24%) |
|  | Unimproved | 5056 (73%) |
|  | Without data | 221 (3%) |
| **Water supply*** | Improved | 5364 (77%) |
|  | Unimproved | 1529 (22%) |
|  | Without data | 64 (0.9%) |
| **Flooring material** | Concrete or tile | 3302 (48%) |
|  | Ground | 3522 (52%) |

*Categorization of water supply and sanitation as improved or unimproved is based in the WHO/UNIFEF JMP definitions (27).
